# Effect of mixing at weaning and nutrient density of the weaner diet on growth performance and welfare of pigs to slaughter

**DOI:** 10.1186/s40813-023-00334-w

**Published:** 2023-08-28

**Authors:** Francesc González-Solé, Jordi Camp Montoro, David Solà-Oriol, José Francisco Pérez, Peadar G. Lawlor, Laura A. Boyle, Edgar Garcia Manzanilla

**Affiliations:** 1 Animal Nutrition and Welfare Service (SNIBA), Department of Animal and Food Science, Autonomous University of Barcelona, Bellaterra, 08193 Spain; 2 Pig Development Department, Animal and Grassland Research and Innovation Centre, Teagasc, Moorepark, Co. Cork, Fermoy, Ireland; 3 School of Veterinary Medicine, University College Dublin, Belfield, 4, Dublin, Ireland

**Keywords:** Behaviour, Chronic aggression, Compensatory growth, Dominance hierarchy, Feed competition, Regrouping, Skin lesions

## Abstract

**Background:**

Mixing pigs at weaning can compromise pig welfare and growth. Therefore, grouping littermates together may allow a diet nutrient and energy density reduction during the nursery period to reduce feed cost without affecting slaughter weight. This study investigated the combined effect of mixing and reducing dietary energy and nutrient density on growth performance, body lesions (BL), and behaviour in pigs from weaning to slaughter.

**Results:**

Forty-eight litters [554 pigs, 11–12 pigs/litter; Danish Duroc × (Large White × Landrace)] were included in the trial. At 28 days of age, pigs were weaned and housed in nursery rooms in litter groups (INTACT, n = 24) or mixed with other litters and grouped by weight to reduce within-pen pig weight variation (MIXED, n = 24). A dietary regimen meeting pigs’ nutritional requirements (CON) and a low-density dietary regimen (LOW; -10% energy and protein) completed a 2 × 2 factorial arrangement (Mixing x Diet, n = 12). On day 74 of age, pigs moved to the grower-finisher accommodation without further mixing and all pigs received the CON dietary regimen. Mixing increased FCR by 4.0% during the nursery period (p = 0.003). Nursery pigs fed LOW experienced a growth retardation which was maintained until slaughter (-2.6 kg slaughter weight; p = 0.025). Initial differences in the coefficient of variation (CV) between MIXED (10.4%) and INTACT (17.6%; p < 0.001) pigs were reduced in CON pens but not in LOW pens (interaction p = 0.025) at the end of the nursery period. MIXED pigs had more fights and BL (p < 0.001) at weaning and showed more aggression (p = 0.003) after being moved to the grower-finisher rooms. At the end of the nursery period, MIXED pigs fed LOW showed the highest number of aggressive behaviours around the feeder (interaction; p = 0.003) and pigs fed LOW showed more damaging behaviour (p < 0.001).

**Conclusions:**

Mixing animals at weaning had limited impact on growth performance but impaired welfare which was aggravated by energy and nutrient reduction in the nursery diet. Decreasing dietary nutrient density in the nursery stage retarded growth, which could not be compensated for during the growing-finishing period.

## Introduction

Mixing or regrouping of pigs is a common management practice used in intensive production systems whereby pigs are sorted into groups by body weight (BW) and/or sex [1]. Unfamiliar pigs are mixed together during all stages of the production cycle (and during transport and lairage) [2] for a variety of reasons including to reduce variation in litter size (i.e. cross fostering), to separate gilts destined as replacements, to adjust group size to the dimensions of the pens, to reduce within-pen bodyweight variation and to achieve more homogenous slaughter weights [3]. However, the initial reduction in BW variability within pens achieved by mixing often increases to values similar to those obtained without mixing the animals [4]. Additionally, social hierarchies need to be established every time unfamiliar pigs are re-grouped. Under intensive commercial practice, pigs establish the dominance hierarchy by fighting and other aggressive strategies [5] that persist for approximately the first 24 h after mixing [6], until a relatively stable hierarchical structure is established [7]. Fights result in skin lesions, have physiological effects, and increase susceptibility to infection due to the immunosuppressive effects of stress [1, 8]. These problems often result in an impairment of growth performance in addition to negative effects on welfare [5, 9–13]. Furthermore, the acute social stress resulting from the mixing of pigs during weaning, combined with the stress of being separated from their mother and the abrupt dietary and environmental change, can have negative consequences for pig health [14]. Once the dominance hierarchy is established after mixing, aggression can continue in the long term due to space restrictions and competition for access to resources [15]. Less attention is paid to the effects of this chronic aggression, although its impact on growth is likely to be significant [15]. Additionally, previous studies from our research group have determined that mixing pigs at the beginning of the growing-finishing period has a lasting negative impact on growth performance. This finding strongly indicates potential long-term welfare implications for the pigs [12, 13].

Some farmers are choosing to lower the energy and nutrient density of nursery diets to cut down on feed costs. This adjustment does not necessarily result in diminished growth performance along the production cycle. In fact, pigs have been observed to show compensatory growth after a period of dietary energy and nutrient reduction, achieved through increased fiber inclusion in their diet [16, 17]. Additionally, pigs show the capacity to increase their feed intake to maintain energy consumption when dietary energy density is reduced [18–20]. While the feed intake capacity of pigs increases as they get older, pigs weighing less than 20 kg may not have the capacity to compensate for severe reductions in energy density [21]. In addition, when feeder space is limited, such as is the case with single-space feeders, the number of skin lesions associated with competition among pigs for access to the feeder increases [22]. Therefore, diluting the energy density of the feed, which will increase the average daily feed intake (ADFI), in combination with a limitation in the number of feeder spaces, increases competition for access to food thereby increases aggression in the longer term i.e. chronic aggression.

Our hypothesis was that allowing piglets to remain with their littermates in a stable social group following weaning would attenuate the impact of the social stress associated with weaning and the subsequent performance of chronic aggressions within the group. Additionally, we expected that providing a low-density diet during the nursery period would have less impact on the growth of pigs kept as intact litters and that any reduction in growth would be compensated for by providing a diet with a higher energy and nutrient density during the growing-finishing period. Thus, the objective of the present study was to determine the impact of mixing and providing low-density nursery diets to pigs on lifetime growth and welfare.

## Methods

### Animals, housing and diet

The present trial was conducted at the Teagasc Pig Research Facility in Fermoy, Co. Cork, Ireland. Two batches of 24 litters with a total of 264 and 288 Danish Duroc × (Large White × Landrace) piglets (docked and un-castrated males), were weaned at 28 days of age and housed in pens of 11 pigs/pen in batch 1 and 12 pigs/pen in batch 2. Pens were allocated to 4 treatments in a 2 × 2 factorial arrangement (Mixing x Diet, n = 12). For the mixing treatment, pigs were in groups of intact litters (LITTER) or mixed with unfamiliar pigs from other litters to reduce the within pen BW variation (MIXED). MIXED pens were composed of big or small mixed-sex pigs. LITTER pens were adjusted to 11 or 12 pigs (depending on the batch) by removing pigs where necessary, preserving a normal distribution of individual BW. For diet, pens were allocated to a dietary regimen meeting the nutritional requirements of the pigs (CON) or a low-density regimen (LOW) with − 10% net energy (NE) and − 10% standard ileal digestible Lysine (SID Lys) of CON. Pigs received a starter diet at weaning for 11 days followed by a link diet from d12 to d22 post-weaning and a weaner diet from d23 to d46 post-weaning when the nursery phase ended (Table 1). Nursery pens were equipped with fully slatted plastic floors (2.5 × 2 m) with automatic environmental control. Each pen had a single-space (33 cm) wet-dry feeder (BA19100, Verba, Netherlands) with inset nipple drinker and a supplementary bowl drinker (SS Drinker, Rotecna, Spain). Nursery pens were enriched with a rubber spiked ball (Easyfix Luna 142, Easyfix, Galway, Ireland).

Pigs were moved to the finisher accommodation at 74 days of age (31.6 ± 3.4 kg BW), keeping the same pen composition as in the nursery rooms. Finisher pens had fully slatted concrete floors (2.4 × 4.2 m) with automatic environmental control, containing one single-space (33 cm) wet-dry feeder (MA19100, Verba, Netherlands) with inset nipple drinker and a supplementary bowl drinker (SS Drinker, Rotecna, Spain). Finisher pens were enriched with a larch wood post. All pigs were fed a single soybean meal-maize-wheat based finisher diet (Table 1) up to target slaughter weight and remained in the facility until the first group of pigs reached 110 kg of BW when all pigs were sent for slaughter. Water and pelleted feed were provided *ad libitum* during the trial.

**Table 1:** Experimental diets offered to the animals included in the trial

	Experimental diets ^1^						
	Starter		Link		Weaner		Finisher
Item	CON	LOW	CON	LOW	CON	LOW	
Ingredients, %							
Barley	5.0	5.0	6.8	59.3	49.6	73.7	41.1
Maize	23.1	37.7	30.0	-	-	-	-
Wheat	-	-	10.0	6.2	21.7	6.0	39.0
Soybean meal 48%	14.3	10.1	18.7	11.3	16.3	14.1	16.5
Full fat soya	13.1	10.0	7.0	10.0	5.0	3.0	1.1
Whey permeate (Lactoflo)	20.0	20.0	15.0	7.5	-	-	-
Skim dried milk	12.5	12.5	5.0	-	-	-	-
Soya oil	8.50	1.38	3.82	1.60	4.00	-	-
Lysine HCl 78.8%	0.622	0.606	0.672	0.736	0.593	0.523	0.427
DL-Methionine	0.362	0.300	0.318	0.296	0.217	0.168	0.100
L-Threonine	0.364	0.329	0.342	0.354	0.271	0.230	0.190
L-Tryptophan	0.140	0.134	0.127	0.113	0.057	0.045	0.022
L-Valine	0.129	0.107	0.126	0.152	0.062	0.017	-
Limestone flour	0.700	0.700	0.750	0.900	1.050	1.050	1.100
Salt	0.300	0.300	0.300	0.300	0.300	0.300	0.300
Mono Dicalcium Phosphate	0.550	0.550	0.700	0.900	0.550	0.550	0.100
Vitamin-Mineral Premix nursery ^2^	0.300	0.300	0.300	0.300	0.300	0.300	-
Vitamin-Mineral Premix finisher ^3^	-	-	-	-	-	-	0.100
Phytase	0.010	0.010	0.010	0.010	0.010	0.010	0.010
Calculated composition							
Dry Matter, %	91.1	90.0	89.4	88.2	87.8	87.2	87.3
Net Energy (NE), MJ/kg	12.06	10.85	10.94	9.85	10.3	9.27	9.80
Ash, %	6.23	5.98	5.65	5.42	4.70	4.76	4.05
Protein, %	20.0	18.0	19.0	17.1	17.7	16.6	16.7
Ether Extract, %	12.18	5.03	6.82	4.73	6.34	2.18	2.66
Neutral Detergent Fiber, %	6.05	6.85	8.08	13.99	13.99	16.15	14.02
Calcium, %	0.819	0.801	0.754	0.767	0.737	0.730	0.652
Total P, %	0.586	0.581	0.567	0.584	0.489	0.497	0.389
Digestible P, %	0.462	0.459	0.423	0.423	0.332	0.336	0.246
Na, %	0.340	0.341	0.263	0.182	0.131	0.131	0.132
Cl, %	0.914	0.916	0.736	0.556	0.352	0.355	0.317
Standard ileal digestible amino acids							
Lys, %	1.528	1.375	1.414	1.272	1.200	1.080	1.000
Met, %	0.680	0.603	0.591	0.513	0.447	0.389	0.324
Cys, %	0.238	0.226	0.258	0.254	0.276	0.264	0.279
Met + Cys, %	0.917	0.825	0.848	0.763	0.720	0.648	0.600
Thr, %	0.993	0.894	0.919	0.827	0.780	0.702	0.671
Trp, %	0.336	0.303	0.311	0.280	0.240	0.216	0.200
Val, %	0.963	0.866	0.891	0.802	0.756	0.680	0.666
Amino acids/Lys ratio							
Lys, %	100	100	100	100	100	100	100
Met, %	45	44	42	40	37	36	32
Cys, %	16	16	18	20	23	24	28
Met + Cys, %	60	60	60	60	60	60	60
Thr, %	65	65	65	65	65	65	67
Trp, %	22	22	22	22	20	20	20
Val, %	63	63	63	63	63	63	67

^1^ Starter diet (d0 to d11 post-weaning, 8.5 to 11.5 kg average BW); Link diet (d12 to d22 post-weaning, 11.5 to 16 kg average BW); Weaner diet (d23 to d46 post-weaning, 16 to 31.5 kg average BW); Finisher diet (growing-finishing period, 31.5 to 116.5 kg average BW).

^2^ Premix provided per kilogram of complete diet: Cu from copper sulphate, 100 mg; Fe from ferrous sulphate monohydrate, 90 mg; Mn from manganese oxide, 47 mg; Zn from zinc oxide, 120 mg; I from potassium iodate, 0.6 mg; Se from sodium selenite, 0.3 mg; vitamin A as retinyl acetate, 2.1 mg; vitamin D3 as cholecalciferol, 25 µg; vitamin E as DL-alpha-tocopheryl acetate, 100 mg; vitamin K, 4 mg; vitamin B12, 15 µg; riboflavin, 2 mg; nicotinic acid, 12 mg; pantothenic acid, 10 mg; choline chloride, 250 mg; vitamin B1, 2 mg; and vitamin B6, 3 mg.

^3^ Premix provided per kilogram of complete diet (Diet 4, finisher): Cu from copper sulphate, 15 mg; Fe from ferrous sulphate monohydrate, 24 mg; Mn from manganese oxide, 31 mg; Zn from zinc oxide, 80 mg; I from potassium iodate, 0.3 mg; Se from sodium selenite, 0.2 mg; vitamin A as retinyl acetate, 0.7 mg; vitamin D3 as cholecalciferol, 12.5 µg; vitamin E as DL-alpha-tocopheryl acetate, 40 mg; vitamin K, 4 mg; vitamin B12, 15 µg; riboflavin, 2 mg; nicotinic acid, 12 mg; pantothenic acid, 10 mg; vitamin B1, 2 mg; vitamin B6, 3 mg.

### Productive performance

Pens of pigs were weighed every two weeks until d151 of age, before they were sent to slaughter. Average daily gain (ADG) was calculated for every 2-week interval. Feed intake was recorded daily at pen level, added for every 2-week period and ADFI was calculated. Feed conversion ratio (FCR) was calculated as $\frac{\text{k}\text{g}\ \text{o}\text{f}\ \text{f}\text{e}\text{e}\text{d}\ \text{c}\text{o}\text{n}\text{s}\text{u}\text{m}\text{e}\text{d}}{\text{B}\text{W}\ \text{g}\text{a}\text{i}\text{n}}$ for each 2-week period. Pigs were also weighed individually at weaning (d28), transfer from nursery to finisher (d74) and before slaughter (d151) to determine the coefficient of variation (CV) within the pen.

### Animal behaviour measurements

Animal behaviour was recorded by direct observation by a blinded observer 1 day post-weaning (d29), three days before transfer to the grower-finisher rooms (d71), one day after the transfer (d75) and at d150, before pigs started to go to slaughter. All pens were observed for three 5 min observations (15 min in total per pen) between 08:00 and 16:00 h. All occurrences of aggression, aggression around the feeder, damaging and sexual behaviour were recorded (Table 2). Observations were equally distributed across pens and time for each recording period.

**Table 2:** Ethogram for the recording of negative behaviours in mixed and intact groups of pigs on two diets. (adapted from O’Driscoll et al., 2013)

	Behaviour	Description
Aggression	Fight	Sustained aggressive biting/pushing by ≥ 2 pigs
	Bite	Biting aggressively at head/body of other pig
	Head knock	Hitting vigorously with head against body/head of other pig
	Parallel pressing	Pressing shoulders against body of other pig, pushing
Aggression around feeder	Displacement from feeder	Pig displaced from feeder by another pig
	Bite at feeder	Biting at body of other pig, at feeder
	Head Knock at feeder	Hitting vigorously with head against body/head of other pig, at feeder
	Climb at feeder	Placing two front hoofs on the body/head of another pig, at feeder
Damaging	Belly nose	Repeated thrusting of snout into belly of another pig
	Ear bite	Ear of other pig in mouth
	Tail bite	Tail of other pig in mouth
Sexual	Sexual mount	Placing two front hoofs on the body/head of another pig

### Body lesion counts

Following the Welfare Quality® criteria [23], the body of the pigs was divided into anterior, mid and posterior part. A body lesion (BL) was defined as either surface penetration of the epidermis or penetration of the muscle tissue [23]. All skin lesions in each location were counted individually as BL, recorded on a check sheet, and summed up to obtain the total number of BL per pig [23]. Lesions arising from damaging behaviour were scored according to severity (ears: 0–4 and tails: 0–3), both scales are described in [24]. Lesions were counted and ears and tails were scored 2 days post-weaning (d30), two weeks later (d42), two days before the transfer to grower-finisher rooms (d72), two days after the transfer (d76), at d144 and at d151, before pigs started to go to slaughter.

### Statistical analyses

All data were analysed in open-source software R v4.0.3 (R Foundation for Statistical Computing, Vienna, Austria). Each pen was considered as the experimental unit for all data analyses. Growth performance data and BL counts were analyzed using general linear mixed models and behaviour data were analyzed using a generalized linear mixed model for a Poisson distribution. The model included mixing, diet and their interaction as fixed effects, and batch as a random effect. The reported *p* values for main effects include the interaction in the model. Growth performance data analysis used initial BW as a covariable while BL and behaviour observations analysis used the BW at the time of the measurement as a covariable. Multiple means comparisons were done using Tukey-Kramer’s correction when the analysis revealed an interaction between mixing and diet. Ear and tail scores did not met the criteria for parametric statistics. Thus, scores were analyzed using Kruskal-Wallis test. Post-hoc comparisons of medians were then conducted using the pairwise Wilcoxon test with multiple testing correction. Differences between treatment groups were calculated as $\frac{\text{G}\text{r}o\text{u}\text{p}\ \text{A}-\text{G}\text{r}\text{o}\text{u}\text{p}\ \text{B}}{\text{G}\text{r}\text{o}\text{u}\text{p}\ \text{B}}$. Results are presented as means and standard error means (S.E.M.) for the general linear mixed models and as medians and interquartile ranges (25th and 75th percentiles) for the nonparametric Kruskal–Wallis test. Alpha for determination of significance and tendencies were 0.05 and 0.10, respectively.

## Results

### Body weight, feed intake and feed efficiency traits

The growth performance results are summarized in Table 3. As there was no Mixing x Diet interaction for the growth performance results, only the main effects of each factor will be described. Mixing did not affect BW, ADG or ADFI during the experimental period (*P* > 0.05) but caused a 4.0% increase in the FCR during the d28 to d74 period (*P* = 0.003). Mixing showed no effect on growth performance during the period from d28 to d151.

**Table 3:** Effect of mixing at weaning or keeping pigs in litter groups and two dietary regimens fed in the nursery period on the productive performance of pigs from weaning to slaughter

	Dietary regimen ^2^							
	CON		LOW			*p* value		
Trait ^1^	Litter	Mixed	Litter	Mixed	SEM^3^	Mixing	Diet	Mixing × Diet
Productive performance								
BW d28, kg	8.38	8.43	8.43	8.43	0.294	0.932	0.936	0.926
BW d74, kg	33.1	32.8	30.1	30.4	0.98	0.832	< 0.001	0.485
BW d151, kg	118.8	117.3	114.6	116.2	2.17	0.933	0.025	0.176
ADFI d28 - d74, kg	0.772	0.791	0.757	0.785	0.0193	0.215	0.534	0.760
ADFI d74 - d151, kg	2.503	2.452	2.410	2.428	0.0512	0.595	0.093	0.300
ADFI d28 – d151, kg	1.855	1.831	1.792	1.814	0.0392	0.970	0.291	0.543
ADG d28 - d74, kg	0.538	0.527	0.475	0.477	0.0128	0.637	< 0.001	0.496
ADG d74 - d151, kg	1.113	1.097	1.097	1.114	0.0188	0.999	0.998	0.204
ADG d28 – d151, kg	0.898	0.884	0.864	0.876	0.016	0.950	0.182	0.416
FCR d28 - d74	1.43	1.51	1.60	1.65	0.033	0.003	< 0.001	0.580
FCR d74 - d151	2.25	2.23	2.20	2.18	0.023	0.367	0.008	0.950
FCR d28 – d151	1.94	1.96	1.97	1.98	0.016	0.442	0.127	0.725

^1^ BW: Body weight; ADG: average daily gain; ADFI: average daily feed intake; FCR: feed conversion ratio.

^2^ CON: dietary regimen meeting pigs’ nutritional requirements; LOW: low-density dietary regime with − 10% of energy and protein of CON during the nursery period, but with the same dietary regimen as CON during the growing-finishing period.

^3^ Standard error of the mean.

^a,b,c^ Within rows, significant differences between groups (P < 0.05).

Pigs fed the LOW diet gained 57 g less per day and had a 2.72 kg lower BW at the end of the nursery period (d74) compared to pigs fed the CON diet (*p* < 0.001), but both treatments had a similar ADFI (*p* > 0.05). As a result, the FCR for LOW pigs deteriorated by 10.3% (*p* < 0.001). During the growing-finishing period, when all animals were offered the same diet, no differences in ADG were observed (*p* > 0.05), but pigs that were fed the LOW diet during the nursery period had a 2.4% better FCR (*p* = 0.008) and tended to have lower ADFI (*p* = 0.093). Pigs from the LOW regime had a 2.65 kg lower slaughter weight than CON pigs (*p* = 0.025).

**Fig. 1:**
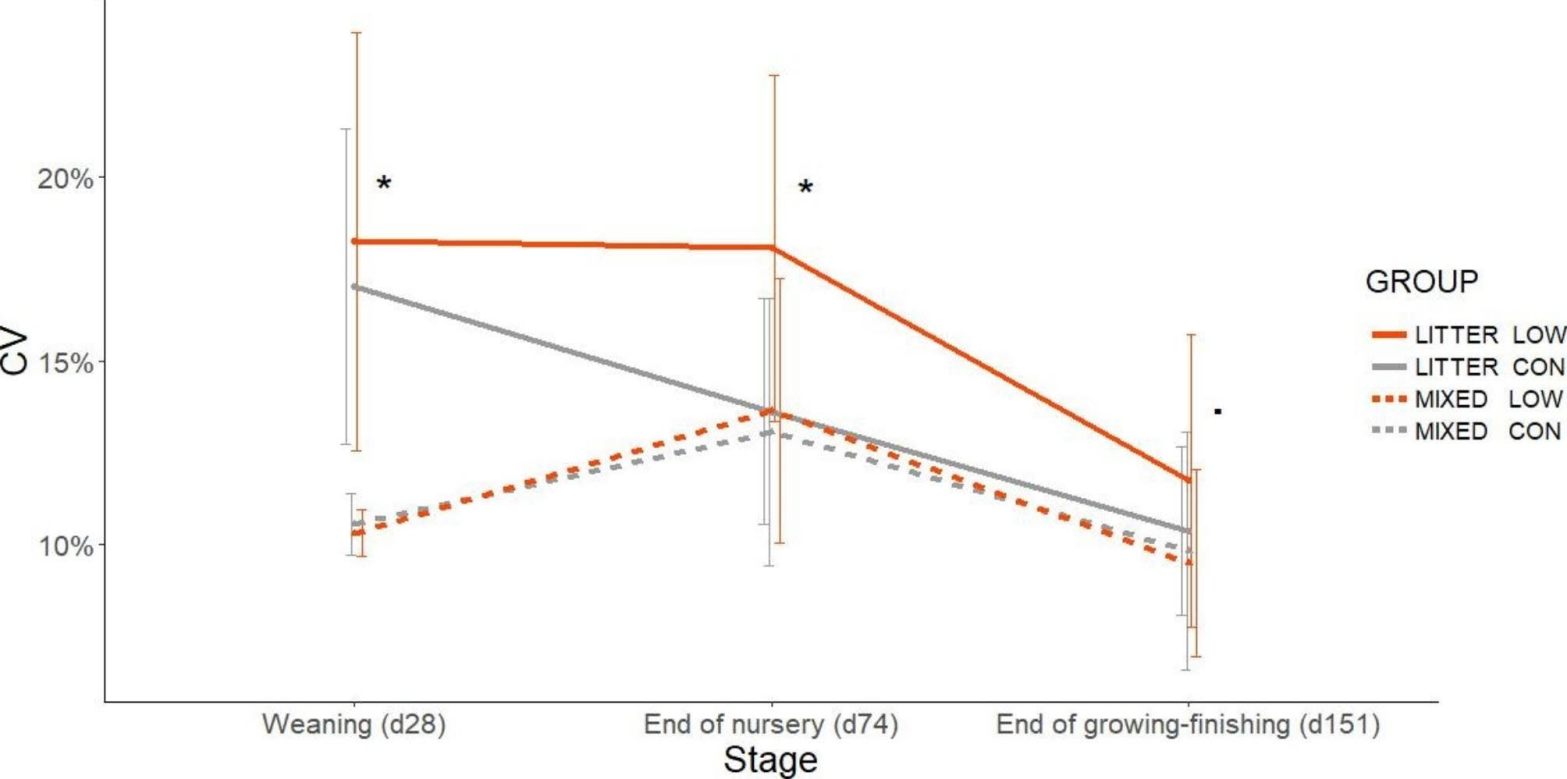
Evolution of the coefficient of variation for within pen pig weight (mean ± SD). Weaning: mixing effect (*p* < 0.001); End of nursery (d74): interaction mixing × diet (*p* = 0.025); End of growing-finishing: mixing effect (*p* = 0.084)

Mixing reduced the coefficient of variation (CV) for pig weight at weaning (d28): 10.4% in MIXED groups compared to 17.6% in LITTER groups (*p* < 0.001; Fig. 1). There was a Mixing x Diet interaction for the CV for pig weight at d74 (*p* = 0.025). The initial difference in CV between MIXED and LITTER groups was reduced in LITTER groups fed the CON diet (13.1% and 13.6%, respectively) but not in those fed the LOW diet (13.6% and 18.1%, respectively). At the end of the growing-finishing period, the differences in CV for BW between MIXED (9.7%) and LITTER pens (11.1%) tended towards significance (*p* = 0.084).

### Body lesion counts and ear and tail lesion scores

The total BL counts and the ear and tail lesion scores are summarized in Table 4. At weaning, MIXED pigs showed 358% more BL and higher ear lesion scores compared to LITTER pigs (*p* < 0.001). At the end of the nursery period, LITTER pigs fed the LOW diet showed higher ear lesion scores than MIXED pigs fed the CON diet (*p* = 0.033). At the beginning of the growing-finishing period, pigs fed the LOW diet during the nursery period showed 14.5% more BL than pigs fed the CON diet (p = 0.009).

**Table 4:** Effect of mixing at weaning or keeping pigs in litter groups and dietary regimen on the total body lesion (BL) counts, ear and tail lesion scores in pigs from weaning to finish

	Dietary regimen ^2^					*p* values			
	CON		LOW			*Kruskal-Wallis*	*GLMM*		
Trait ^1^	Litter	Mixed	Litter	Mixed	SEM ^3^	Group	Mixing	Diet	Mixing × Diet
**Weaning, d30**									
Total BL	11.6	36.8	9.9	42.4	2.92	-	< 0.001	0.523	0.161
Ear score	0.13 ^b^ (0.06–0.34)	0.58 ^a^ (0.39–1.09)	0.04 ^b^ (0–0.21)	0.86 ^a^ (0.74–1)	-	< 0.001	-	-	-
Tail score	0 (0–0)	0 (0–0)	0 (0–0)	0 (0–0)	-	1.000	-	-	-
**Nursery, d42**									
Total BL	4.6	4.2	4.5	3.4	0.84	-	0.296	0.891	0.790
Ear score	0 (0–0)	0 (0–0)	0 (0–0.02)	0 (0–0)	-	0.896	-	-	-
Tail score	0 (0–0)	0 (0–0)	0 (0–0)	0 (0–0)	-	0.563	-	-	-
**End of nursery, d71**									
Total BL	24.3	22.8	23.0	24.7	1.38	-	0.908	0.091	0.259
Ear score	0 ^ab^ (0–0)	0 ^b^ (0–0)	0.042 ^a^ (0–0.26)	0 ^ab^ (0–0.02)	-	0.033	-	-	-
Tail score	0 (0–0)	0 (0–0)	0 (0–0)	0 (0–0)	-	0.790	-	-	-
**Beginning of growing-finishing, d76**									
Total BL	23.5	26.1	27.2	29.6	1.96	-	0.155	0.009	0.517
Ear score	0 (0–0)	0 (0–0)	0 (0–0.13)	0 (0–0)	-	0.343	-	-	-
Tail score	0 (0–0)	0 (0–0)	0 (0–0)	0 (0–0)	-	0.563	-	-	-
**End of growing-finishing, d151**									
Total BL	19.8	18.8	19.5	18.1	1.31	-	0.311	0.760	0.832
Ear score	0 (0–0)	0 (0–0)	0 (0–0)	0 (0–0)	-	0.392	-	-	-
Tail score	0 (0–0)	0 (0–0)	0 (0–0)	0 (0–0)	-	0.563	-	-	-

^1^ Total body lesions (BL): Sum of body lesions (BL) from the anterior, middle, and posterior body sections in each pig expressed as mean average; Ear score: ear lesions on a scale 0–4. Tail score: tail lesions on a scale 0–3. Ear and tail scores are expressed as medians with interquartile ranges (md (Q25–Q75)). Values of ear and tail scores within a row with different letters significantly differ (a,b: p < 0.05).

^2^ CON: dietary regimen meeting pigs’ nutritional requirements; LOW: low-density dietary regime with − 10% of energy and protein than CON during the nursery period, but with the same dietary regimen as CON during the growing-finishing period.

^3^ Standard error of the mean.

### Pig behaviour

Behaviour data are detailed in Table 5. After weaning, the number of aggressive behaviours and aggressive behaviours around the feeder showed an interaction between mixing and diet (*p* < 0.001; *p* = 0.005). MIXED pigs fed the LOW diet performed the highest number of aggressive behaviours, followed by LITTER pigs fed the LOW diet, with MIXED pigs fed the CON diet showing fewer aggressive behaviours than pigs in pens fed the LOW diet. LITTER pigs fed the CON diet showed fewer aggressive behaviours around the feeder at weaning than the rest of groups (p < 0.005 interaction). MIXED pigs showed more damaging (*p* = 0.042) and sexual behaviour (*p* = 0.039) towards other pen mates than LITTER pigs.

**Table 5:** Effect of mixing or being in litter groups at weaning and two dietary regimens on the occurrence of different negative behaviours in pigs from weaning to finish

	Dietary regimen ^1^							
	CON		LOW			*p* value		
Trait ^1^	Litter	Mixed	Litter	Mixed	SEM^3^	Mixing	Diet	Mixing × Diet
**Post-weaning, d29**								
Aggression	3.79 ^bc^	2.96 ^c^	4.25 ^b^	7.66 ^a^	1.159	0.005	< 0.001	< 0.001
Feeder	0.67 ^b^	2.00 ^a^	1.88 ^a^	2.04 ^a^	0.597	0.024	0.037	0.005
Damaging	2.33	2.58	2.29	3.46	0.455	0.042	0.191	0.254
Sexual	0.75	1.08	0.46	1.21	0.449	0.039	0.674	0.184
**End of nursery, d70**								
Aggression	3.25 ^a^	1.58 ^b^	2.83 ^ab^	3.17 ^a^	0.685	0.195	0.257	0.023
Feeder	2.75 ^bc^	1.08 ^c^	3.92 ^ab^	4.75 ^a^	1.048	0.515	< 0.001	0.003
Damaging	3.66	3.5	5.42	6.5	0.978	0.471	< 0.001	0.398
Sexual	2.08	1.92	1.17	1.67	0.528	0.667	0.131	0.331
**Beginning of growing-finishing, d75**								
Aggression	6.17	8.58	4.25	7.67	1.720	< 0.001	0.066	0.292
Feeder	3.58	3.75	3.33	2.92	1.146	0.814	0.313	0.564
Damaging	4.08	4.58	5.42	3.75	0.847	0.304	0.666	0.075
Sexual	2.33	1.25	1.25	1.17	0.467	0.110	0.110	0.257
**End of growing-finishing, d150**								
Aggression	2.33 ^b^	3.92 ^ab^	4.92 ^a^	2.58 ^b^	0.911	0.445	0.286	< 0.001
Feeder	2.58	2.33	2.17	1.58	0.696	0.263	0.181	0.574
Damaging	3.00	1.75	3.83	2.08	0.597	< 0.001	0.198	0.814
Sexual	0.00	0.33	0.75	0.58	0.227	0.600	0.372	0.986

^1^ Occurrence of different behaviours for a period of 15 min by pen.

^2^ CON: dietary regimen meeting pigs’ nutritional requirements; LOW: low-density dietary regime with − 10% of energy and protein than CON during the nursery period, but with the same dietary regimen as CON during the growing-finishing period.

^3^ Standard error of the mean.

^a,b,c^ Within rows, significant differences between groups (P < 0.05).

At the end of the nursery period (d70), MIXED pigs fed the CON diet showed fewer aggressive behaviours than MIXED pigs fed the LOW diet and LITTER pigs fed the CON diet (interaction; p = 0.023). The analysis also revealed an interaction in the number of aggressive behaviours around the feeder (*p* = 0.003): MIXED pigs fed the LOW diet performed more aggression at the feeder than pigs fed the CON diet and pigs in pens fed the LOW diet performed more of these behaviours compared to MIXED pigs fed the CON diet. Pigs fed the LOW diet showed more damaging behaviours than pigs fed the CON diet (p < 0.001).

At the beginning of the growing-finishing period, MIXED pigs showed more aggressive behaviours (p < 0.001). Pigs in pens fed the LOW diet showed a tendency to perform fewer aggressive behaviours (p = 0.066).

At the last observation before slaughter (d150), the number of aggressive behaviours showed an interaction between mixing and diet (p < 0.001). LITTER pigs fed the LOW diet showing more aggressive behaviours than LITTER pigs fed the CON diet and MIXED pigs fed the LOW diet. In addition, LITTER pigs performed more damaging behaviours than MIXED pigs.

## Discussion

### Growth performance

Re-grouping of pigs at weaning requires the establishment of a new social order [6] through aggressive behaviour, which results in skin lesions, psychological stress [1] and compromised productive performance [25]. We hypothesized that apart from the initial fights for hierarchy establishment at re-grouping, MIXED pigs would show a poorer social stability along the production cycle, displaying more conflicts and chronic aggression when dealing with a nutritionally low-density diet. Pigs in MIXED groups showed higher levels of aggression and had more body lesions post-weaning compared to pigs kept in LITTER groups. Additionally, the results support that a low-density diet interacts with mixing at weaning, as aggression was more prevalent in MIXED groups fed the LOW diet. In line with previous reports [18–21], nursery pigs fed a low energy and protein diet were unable to increase their voluntary feed intake to compensate for the reduced dietary nutrient and energy density and so their growth was reduced. Consequently, the FCR of pigs fed the LOW diet was 10.3% poorer during the nursery period.

Although pigs expend a high amount of energy fighting to establish the social order [9] and the associated stress response is energy costly, mixing did not affect weight gain of pigs. Mixing caused a slight numerical increase in feed intake, which resulted in a worsened FCR during the nursery period. These findings are in contrast to previous studies that identified a negative effect of regrouping involving an impairment of growth. Camerlink et al. (2021) [25] reported poorer growth during the first week post-weaning after mixing weaned pigs compared to keeping them in littermate pens. Additionally, other authors associated the regrouping of pigs at the beginning of or during the growing-finishing period with a reduction in growth [5, 9–13]. It may be that under the conditions of the current study that the aggression exhibited due to mixing was not sufficiently intense to compromise growth, but this is difficult to confirm since most previous studies did not include behavioural data.

One of the goals of mixing pigs at weaning is to reduce the variability in pig BW within the pen. Previous studies observed that while the co-efficient of variation (CV) for within pen pig weight was initially reduced due to mixing, it then gradually increased until the end of the growing and finishing period when mixed groups had similar CV for pig weight as pen-groups of littermates (i.e. not remixed) [4, 26]. Indeed, Tindsley and Lean (1984) [26] proposed that a certain degree of variation in BW between individuals within a group is a necessary component of group social dynamics and that groups of animals will therefore tend towards such variation. Accordingly, the CV for pig weight increased among pigs in MIXED groups and converged with the CV in BW of LITTER groups fed the CON dietary regime. However, the CV in BW of LITTER groups fed the LOW diet did not decrease to the levels observed in the other three treatment groups. This result is probably explained by the fact that the lighter pigs in LITTER pens had reduced feed intake capacity. Therefore, they could not ingest sufficient nutrients and energy from the low-density diet to catch up the heaviest littermates. In the same line, Douglas et al. (2014) [27] found that light birth weight pigs benefited more from a high specification post-weaning diets than their normal birthweight counterparts. In growing pigs, Hastad et al. (2020) [28] demonstrated that increasing the dietary energy density for pigs from 30 kg BW mainly favored the growth of the lighter half of the pigs and reduced the within-pen CV of bodyweight at slaughter. Aymerich et al. (2022) [29] also showed that severely limiting dietary SID Lys:NE below nutritional requirements can negatively affect the within-pen CV of pig weight of growing pigs (28–63 kg BW), mainly because the dietary challenge restricted the growth of the lightest pigs. This interaction between the initial BW homogeneity of the pen and dietary regime density was also observed by Magowan et al. (2011) [30]. These authors observed the highest within-pen CV for ADG when they provided a low energy density dietary regime to pigs heterogeneously grouped from weaning to 20 weeks of age, while the lowest CV for ADG was observed when uniformly grouped pigs were fed an energy and nutrient-rich dietary regime.

At the beginning of the growing-finishing period, all treatment groups were moved to finisher accommodation without further mixing and all groups were fed an amino-acid and energy rich finisher diet. For the rest of the trial, MIXED and LITTER pens did not show any differences in growth parameters. This is in contrast with the results of Jones et al. 2011 [31], who reported improved growth during the growing-finishing period in mixed groups of pigs that included full siblings compared to those that did not, although contrary to our study, these pigs underwent another regrouping at the beginning of this growing-finishing period. After providing a growing-finisher diet to meet the dietary requirements of all pigs during the realimentation period, pigs that were fed the LOW diet during the nursery period exhibited the same BW gain and tended to show a lower ADFI than those fed the CON regimen during the nursery period, which translated into a lower FCR for the former. The improved efficiency of pigs fed the LOW diet during the growing-finishing period can be attributed to two potential factors. First, their lower initial BW at the start of this period may have played a role, as pigs generally become less efficient as they grow [32]. Second, this finding could indicate a growth compensation mechanism exhibited by the pigs following the reduction in dietary density [33].

Previous reports showed that pigs can exhibit complete compensatory growth during the recovery period by increasing feed intake after a period of reducing dietary energy and nutrient density [16, 17]. In the present trial, animals fed the LOW diet were still 2.6 kg lighter at slaughter. Menegat et al. (2020) [33] suggested that a recovery period of > 55–60% of the overall period (63% in the present study) was long enough for complete compensatory growth. However, in this trial the nutrient limitation affected the pigs during an early stage of growth when they were still partially growing through cell proliferation [34], potentially influencing the final cell number and limiting the potential for compensatory growth.

In addition, the dietary change at transfer to growing finishing accommodation reduced the within-pen variation in BW of the LITTER pigs that had been fed the LOW diet during the nursery period. By the end of the study, within pen variation in pig weight was no different to that of the other groups, most probably because the lighter pen-mates had the opportunity to show some degree of compensatory growth.

### Behaviour observations and lesion scores

Establishment of the dominance hierarchy in mixed groups was reflected in an increased number of aggressive behaviours, aggressive behaviours around the feeder, body lesions (358% increase) and ear injuries relative to pigs kept in litter groups in the 24 to 48 h post weaning. The number of aggressive behaviours, including aggression at the feeder, showed an interaction with the diet, which is difficult to explain given the short time it was offered to the pigs before the behavioural observations were performed and the low feed intake in the first hours post-weaning. However, it has been suggested that after abrupt weaning, piglet sill have dependence on high oleic acid lipids as found in milk and prefer feeds with a higher lipid inclusion than typically offered in weaner diets [35]. Therefore, the superior fat content of the CON diet (12.18%) compared to the LOW diet (5.03%) might have helped piglets to cope better with the transition from sow’s milk to the starter diet, which might have reduced the stress level and the social tension among pigs. However, the dietary regimen had no effect on the number of body lesions which are a good proxy for aggressive behaviour on commercial farms [36, 37]. In the current study, the body lesions probably showed a more realistic picture of the aggressive interactions that occurred after mixing the pigs, as aggressive behaviour caused by mixing generally subsides within 24 h [6], while in the current study behaviour observations were only performed after this time.

During the same post-weaning observation, LITTER pigs showed a lower frequency of damaging oral behaviour towards other pigs, including belly nosing, tail biting and ear biting than MIXED pigs. The expression of damaging behaviours likely reflected the more stressed state of the mixed animals after weaning [38]. Mixed pigs showed more ear lesions, although, in this stage, they were more likely caused by aggression than by oral ear manipulation. Tail scores were non-existent at the post weaning inspection, indeed they were low throughout the study and did not differ between treatments probably reflecting the fact that the pigs were docked [39]. Additionally, mixed groups showed more mounting behaviour than litter groups after weaning. Mounting may occur when the dominant pig settles its rank [40] or to demonstrate the dominance status [41] and probably was part of the set of behaviours associated with establishment of the dominance hierarchy. These results are in line with Camerlink et al. (2021) [25], who also identified an increase in sexual mounting in regrouped pigs at weaning. However, they did not observe increases in the performance of damaging behaviour in response to mixing, probably because of the large between-pen variability found in their observations.

There is normally a reduction in aggressive behaviour between mixed pigs once a new stable social order is established, approximately two weeks after mixing [5]. In agreement, the number of body lesions did not differ between treatment groups two weeks after weaning in the current study. However, in socially stable groups chronic aggression may persist, refining previously established social relationships and are often triggered during competition for limited resources [42]. Prior studies revealed a long-term negative effect of mixing growing-finishing pigs on their performance [12, 13], suggesting that there may be long-term implications for their welfare. While there was an initial increase in the number of body lesions immediately after mixing, this increase in body lesions was not followed by an effect on performance which appeared weeks later [12]. To gain further insights into this finding, in the present trial, we incorporated behaviour observations to uncover any potential link between mixing and chronically altered behavior that could elucidate the adverse performance outcomes observed in the earlier studies. In the present study, the number of aggressive behaviours and aggression around the feeder at the end of the nursery period increased where the LOW dietary regimen was fed to MIXED groups of pigs. Pigs fed the LOW diet, especially the ones that were mixed performed more aggression associated with the feeder than pigs fed the CON diet at the end of the nursery period. At the time of the observations (d70), pigs were close to reaching 30 kg BW and probably already had some capacity to increase their physical feed intake to increase their energy and amino acid intake on the LOW regimen. The low-density diet, especially when provided via a single-spaced feeder, might have stimulated increased competition for access to feed and, consequently, increased the number of aggressive behaviours around the feeder. This could have increased the risk of conflicts given the limited feeder space [43], resulting in high levels of stress and aggression [44]. The level of aggression and aggression around the feeder was lowest in MIXED pigs fed the control diet. However, the nutritional treatment only had a significant effect in pigs in the MIXED pens. This result suggests that when pigs are fed the control diet, mixed animals might perform less chronic aggression compared to groups of littermates. However, when they are fed a reduced nutrient and energy density diet and potentially face an increased competition for access to feed, they may display more chronic aggressions compared to groups of littermates. Nevertheless, the differences in the aggressive behaviour observed among groups were not reflected in the number of skin lesions.

Pigs fed the LOW dietary regimen showed more damaging behaviour towards pen mates at the end of nursery period, which might reflect that these pigs were nutritionally limited. When growth or immune functioning are limited by nutrient availability, pigs can increase their foraging behaviour to satisfy their nutritional needs [45]. If rooting substrates are not sufficiently available, pigs can redirect their foraging and exploratory behaviour to nosing, chewing, or sucking certain body parts of their pen mates, which could end up in vigorous biting leading to wounds [46]. Others have described an increase in the occurrence of damaging behaviours such as ear and tail biting when protein requirements were not fulfilled [45, 46]. Our findings revealed that pigs in LITTER pens fed the LOW dietary regime exhibited higher ear lesion scores compared to MIXED pigs fed the CON diet at this stage. However, it is important to note that the overall scores were generally low. The observed difference in scores was primarily influenced by two pens where an outbreak of ear attacks occurred performed by a single pig in each case. Therefore, it is likely that the role of the experimental treatments in the higher ear lesion scores was probably minimal in the current study.

When pigs were moved to the grower-finisher rooms, there was a general increase in aggressive behaviour. This effect was already described by Moore et al. (1994) [47] who found that pigs from static groups fought more after rehousing than pigs of similar size housed in dynamic groups. Nevertheless, this increase in aggressions was minor in the pigs kept in litter groups, suggesting they preserved a better social group stability than those in MIXED groups. Despite this, the total skin lesion counts at the beginning of growing-finishing period did not show a difference between pigs in MIXED and LITTER pens.

After substituting the low-density nursery diet by the growing-finishing diet with a higher nutritional density, the differences between groups in aggression around the feeder disappeared. Even pigs that received the LOW diet during nursery period showed a tendency to perform less aggression after the dietary change. This emphasizes the significant role that competition for feed played in the development of chronic aggression. However, pigs previously fed the LOW dietary regime during the nursery period showed more skin lesions. The explanation for this contrasting finding may be that these lesions possibly resulted from the aggression around the feeder performed during the last days of nursery phase, which was higher in the pigs fed the LOW diet.

At the end of growing-finishing period, there was an interaction between mixing and diet for the counts of aggressive behaviour. LITTER pigs fed the LOW diet showed more aggressive behaviours than LITTER pigs fed the CON diet and MIXED pigs fed the LOW diet. LITTER pigs also performed more damaging behaviours than MIXED pigs. These differences are difficult to interpret but given the passage of time since weaning/mixing and the dietary challenge it is likely that these findings are not biologically relevant. In addition, the lesion scoring did not corroborate these behavioural differences between treatments.

## Conclusions

Overall, the practice of mixing pigs at weaning triggered fights for hierarchy re-establishment and increased stress in pigs immediately after weaning as reflected by the increased amount of damaging behaviour. Chronic aggression was reduced at the end of the nursery period by mixing at weaning, but not when a low nutrient and energy density diet was fed. Furthermore, chronic aggression was increased in mixed pigs after being moved to the grower-finisher accommodation. Thus, although mixing animals at weaning had a limited impact on pig growth, it had a detrimental effect on welfare and should be avoided, especially when pigs are fed low nutrient and energy density diets.

Provision of a low-density diet during the nursery period caused a growth retardation that could not be compensated for during the growing-finishing period. In addition, it increased the variation in BW in litter-mate pens, possibly because lightweight pigs within the pen were especially affected by the low-density dietary regimen. Furthermore, diets in this experiment were fed from single-space feeders which likely aggravated competition for feed and contributed to the increase seen in the performance of damaging behaviour at the end of the nursery period when the low-density diet regimen was fed.

## Data Availability

The datasets used and analyzed during the current study are available from the corresponding author on reasonable request.

## Abbreviations

ADG: average daily gain
ADFI: average daily feed intake
BW: Body weight
BL: Body lesions
CON: Dietary regiment meeting the nutritional requirement of the pigs
CV: Co-efficient of variation
FCR: Feed conversion ratio
LITTER: Pigs kept in groups of littermates at weaning
LOW: Low density regiment with with − 10% net energy (NE) and − 10% standard ileal digestible Lysine (SID Lys) of CON
MIXED: Pigs mixed with unfamiliar pigs from other litters at weaning to reduce the within pen BW variation
NE: Net energy
SID: Standard ileal digestible
